# Full Genome Sequencing of Corriparta Virus, Identifies California Mosquito Pool Virus as a Member of the *Corriparta virus* Species

**DOI:** 10.1371/journal.pone.0070779

**Published:** 2013-08-27

**Authors:** Manjunatha N. Belaganahalli, Sushila Maan, Narender S. Maan, Kyriaki Nomikou, Marc Guimera, Joe Brownlie, Robert Tesh, Houssam Attoui, Peter P. C. Mertens

**Affiliations:** 1 The Vector-Borne Viral Diseases Programme, The Pirbright Institute, Pirbright, Woking, Surrey, United Kingdom; 2 College of Veterinary Sciences, LLR University of Veterinary and Animal Sciences, Hisar, Haryana, India; 3 Department of Pathology and Infectious Diseases, Royal Veterinary College, North Mymms, Hatfield, Herts, United Kingdom; 4 Department of Pathology, University of Texas Medical Branch, Galveston, Texas, United States of America; Columbia University, United States of America

## Abstract

The species *Corriparta virus* (CORV), within the genus *Orbivirus*, family *Reoviridae*, currently contains six virus strains: corriparta virus MRM1 (CORV-MRM1); CS0109; V654; V370; Acado virus and Jacareacanga virus. However, lack of neutralization assays, or reference genome sequence data has prevented further analysis of their intra-serogroup/species relationships and identification of individual serotypes. We report whole-genome sequence data for CORV-MRM1, which was isolated in 1960 in Australia. Comparisons of the conserved, polymerase (VP1), sub-core-shell ‘T2’ and core-surface ‘T13’ proteins encoded by genome segments 1, 2 and 8 (Seg-1, Seg-2 and Seg-8) respectively, show that this virus groups with the other mosquito borne orbiviruses. However, highest levels of nt/aa sequence identity (75.9%/91.6% in Seg-2/T2: 77.6%/91.7% in Seg-8/T13, respectively) were detected between CORV-MRM1 and California mosquito pool virus (CMPV), an orbivirus isolated in the USA in 1974, showing that they belong to the same virus species. The data presented here identify CMPV as a member of the *Corriparta virus* species and will facilitate identification of additional CORV isolates, diagnostic assay design and epidemiological studies.

## Introduction

Corriparta viruses are mosquito-borne arboviruses, classified within one of the 22 virus species currently recognised within the genus *Orbivirus*, family *Reoviridae*. Currently there are also 15 ‘unclassified’ orbiviruses in the genus, which may represent additional species [1]–[6]. The orbivirus genome is composed of 10 segments of linear dsRNA, packaged as one copy of each segment within each of the non-enveloped icosahedral virus particles. The intact virion is composed of three concentric protein shells (the ‘outer-capsid’, ‘core-surface layer and the ‘subcore-shell’). Orbiviruses are transmitted by ticks or hematophagus-insect vectors (including *Culicoides*, mosquitoes or sand flies) and collectively have a wide host-range that includes both domesticated and wild ruminants, equids, camelids, marsupials, sloths, bats, birds, large canine and feline carnivores, and humans [2], [7]–[9].

The species *Corriparta virus* currently contains six distinct viruses, that are identified as: corriparta virus MRM1 (CORV-MRM1); CS0109; V654; V370; Acado virus; and Jacareacanga virus [2]. The structural and chemical properties of the corriparta viruses are similar to those of other orbiviruses [2]. They are sensitive to low pH and heat, and can be modified by treatment with trypsin or chymotrypsin [10]. They have also been shown to multiply in mosquitoes after intra-thoracic inoculation [10].

Members of the *Corriparta virus* species/serogroup have been detected in Australia, Africa and South America [11]). They have been isolated from wild birds, and neutralizing antibodies were found in wild and domestic birds, cattle, marsupials, horses and man [12]–[15]. Corriparta virus MRM1 was isolated in 1960, from *Culex* mosquitoes, as well as from *Aedeomyia catasticta*, a rare mosquito species collected near Mitchell River in North Queensland, Australia. Subsequently, strains CS0109, V654 and V370 were also isolated in Australia [2], [8], [15], [16]. Acado virus and Jacareacanga virus were isolated from pools of *Culex* mosquitoes collected in Ethiopia and Brazil during 1963 and 1975 respectively [8], [11].

The International Committee on Taxonomy of Viruses (ICTV) has agreed ‘polythetic’ definitions for individual virus species [17]. The ability to exchange genome segments with other viruses belonging to the same virus species by ‘reassortment’ is recognised as the primary determinant of *Orbivirus* species [2], [7]. However, in the absence of data concerning their compatibility for reassortment, the members of individual species can be identified by other ‘polythetic’ parameters that include similarities in RNA and protein sequences, their RNA-segment size distribution (reflected by their migration patterns - electropherotype) during agarose gel electrophoresis [AGE], host and/or vector range, the clinical signs of infection, and serological relationships [2], [7], [18]–[20].

The members of the different *Orbivirus* species were originally identified as belonging to distinct ‘serogroups’, based on their cross-reactivity in ‘group-specific’ serological assays that include complement fixation (CF) tests, group-specific ELISA, or agar-gel-immuno-diffusion (AGID) tests, most of which target outer-core protein VP7(T13) [2], [7], [21]. The corriparta viruses were initially grouped primarily on the basis of CF tests [8], [22]. However, a lack of neutralization assays has prevented further analysis of their intra-serogroup serological-relationships and the identification of distinct serotypes.

Recently, full genome sequencing and phylogenetic analyses have been used to determine the genetic relatedness and taxonomic status of individual isolates belonging to different *Orbivirus* species, including *Bluetongue virus* (BTV); *African horsesickness virus* (AHSV) and *Epizootic hemorrhagic disease virus* (EHDV) [1], [23]–[26]. These sequence data have supported development of faster and more reliable, virus-species/serogroup, and virus-serotype specific diagnostic assays, using both conventional and real-time RT-PCRs [27]–[31]. Comparisons of nucleotide (nt) and amino acid (aa) sequence data also provide a basis for grouping of orbivirus isolates into topotypes and for molecular epidemiology studies [23], [25], [32], [33]. However, full-genome sequence data are currently available for representatives of only 11 of the recognized *Orbivirus* species [24] (accession numbers given in Table S1). Additional partial-sequences are available for the highly conserved genome-segment encoding the subcore ‘T2’ protein (VP3 of BTV) of some *Orbivirus* species, including *Warrego virus* (WARV), *Wallal virus* (WALV), *Wongorr virus* (WGRV) and CORV [32] (Table S1).

‘California mosquito pool virus’ (CMPV) was isolated in 1974 from pooled *Culex tarsalis* mosquitoes collected as part of an infectious agent surveillance program conducted by The California Department of Public Health [5]. Partial sequences for genome segments 2, 4, 6, 7 and 9 from CMPV (accession numbers EU789391 to EU789395) were compared to available data for other orbiviruses, suggesting that CMPV might represent a novel virus species [5]. However, the lack of reference sequences for representatives of all *Orbivirus* species, made it impossible to confirm the taxonomic status and species identity of CMPV at that time.

We report the full genome sequence of CORV–MRM1 (AUS1960/01). Comparisons of nucleotide (nt) and deduced amino acid (aa) sequences for the conserved polymerase ‘VP1(Pol)’, subcore-shell ‘T2-protein’, and outer-core ‘T13-protein’, to data published for other orbiviruses, indicate that CORV and CMPV belong to the same species - *Corriparta virus*.

## Results

### Virus propagation and dsRNA ‘electropherotype’

**Figure 1:**
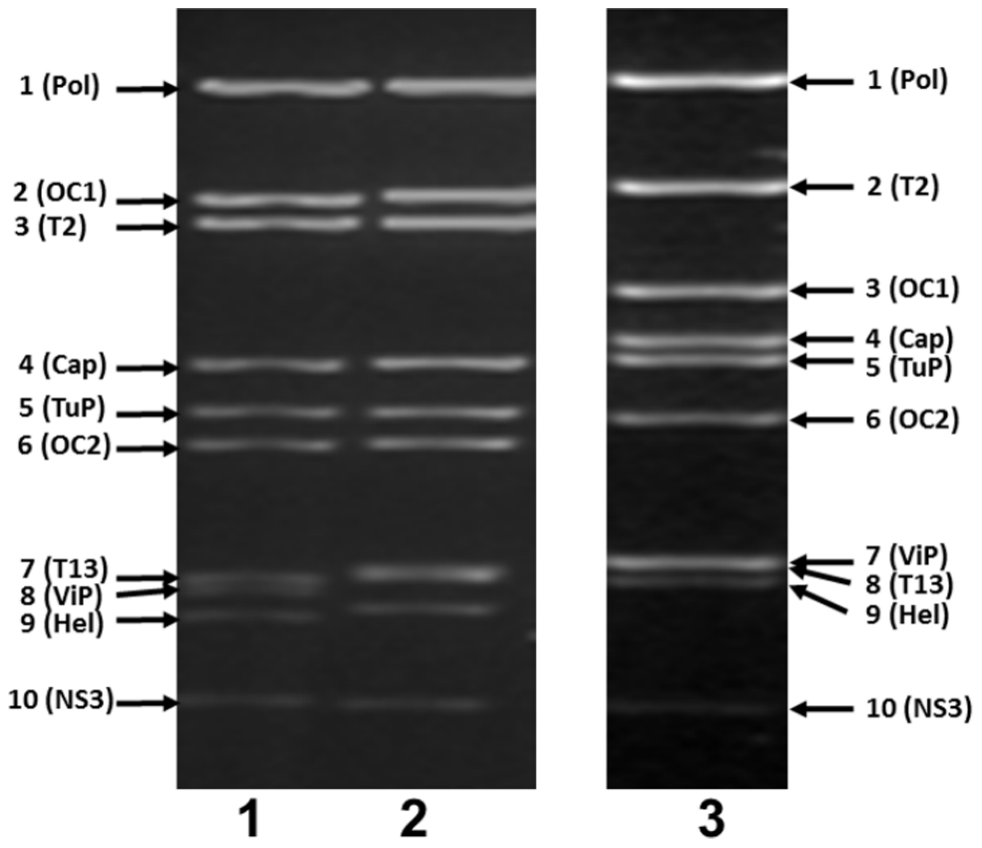
Agarose gel (1%) electrophoresis for dsRNAs of CORV, BTV and EHDV. The purified genomic RNA of LIB2007/07 (a western-topotype isolate of BTV serotype 1 (BTV-1w)) - lane 1: AUS1982/05 (an eastern-topotype isolate of EHDV serotype 8 (EHDV-8e)) - lane 2: and CORV-MRM1 (AUS1960/01) - lane 3, were analysed by 1% agarose gel electrophoresis (AGE), then stained with ethidium bromide, visualised and photographed under UV illumination. The genome segment numbers (encoded proteins) are indicated at the side of the gel.

CORV–MRM1 induced severe cytopathic effect (CPE) in BHK cell monolayers, by 48–72 hours post infection (pi). The viral dsRNA was purified from infected cell-cultures and analysed by 1% agarose gel electrophoresis (AGE) (Figure 1). CORV Seg-3 (encoding the larger outer capsid protein VP3(OC1)) migrates close to the medium sized genome-segments (Seg-4, Seg-5 and Seg-6) giving a 2-4-4 (2-4-3-1) migration pattern (Figure 1). This contrasts with the 3-3-4 (3-3-3-1) pattern that is more typical of the *Culicoides* borne orbiviruses (as illustrated for BTV-1w [LIB2007/07] and EHDV-8e [AUS1982/05]).

### Characterisation and coding assignments of CORV genome segments

Sequences for Seg-1 to Seg-10 CORV-MRM1 (AUS1960/01) have been deposited in the GenBank with accession numbers KC853042 to KC853051, respectively. They range from 3,925 bp to 790 bp (encoding proteins of 1,290 aa to 108 aa) with a total length of 19,093 bp, (Table 1). The different genome segments of CORV-MRM1 all share six fully conserved nucleotides at their 5′ ends, and 10 at their 3′ ends (+ve: 5′-GUAUAG………..CAAAGGAUAC-3′). Two terminal nucleotides at the 5′ end and three nucleotides at the 3′ end (5′-GU……UAC-3′) are characteristic of the genus *Orbivirus* and the first and last two nucleotides represent inverted complements (http://www.reoviridae.org/dsRNA_virus_proteins/CPV-RNA-Termin.htm).

**Table 1:** Characteristics of Corriparta virus (CORV-MRM1) (AUS1960/01) genome segments (dsRNA) and their encoded proteins.

Seg No.	Seg size (bp)	G+C content (%)	Protein encoded (structure/putative function)	ORFs bp (including stop codon)	5′NCR	3′NCR	No. of amino acids	Predicted protein molecular mass (*kDa*)	5′ Termini	3′ Termini	Accession numbers
**1**	3,925	41.66	VP1 (Pol)	12-3884	11	44	1290	147.07	**5′-GUAUAG**G….	….C**CAAAGGAUAC-3′**	KC853042
**2**	2,952	43.43	VP2 (T2)	33-2894	32	61	953	108.75	**5′-GUAUAG**G….	….C**CAAAGGAUAC-3′**	KC853043
**3**	2,284	42.43	VP3 (OC1)	20-2236	19	51	738	84.81	**5′-GUAUAG**G….	….C**CAAAGGAUAC-3′**	KC853044
**4**	2,032	43.11	VP4 (Cap)	12-1943	11	92	643	74.83	**5′-GUAUAG**A….	….U**CAAAGGAUAC-3′**	KC853045
**5**	1,936	46.33	NS1 (TuP)	20-1789	19	150	589	68.61	**5′-GUAUAG**A….	….U**CAAAGGAUAC-3′**	KC853046
**6**	1,683	46.11	VP5(OC2)	56-1639	55	47	527	57.7	**5′-GUAUAG**A….	….U**CAAAGGAUAC-3′**	KC853047
**7**	1,187	46.5	NS2 (ViP)	26-1141	25	49	371	41.86	**5′-GUAUAG**A….	….U**CAAAGGAUAC-3′**	KC853048
**8**	1,176	47.62	VP7 (T13)	17-1081	16	98	354	38.96	**5′-GUAUAG**A….	….U**CAAAGGAUAC-3′**	KC853049
**9**	1,128	47.16	VP6 (Hel)	49-1083	48	48	344	36.85	**5′-GUAUAG**A….	….U**CAAAGGAUAC-3′**	KC853050
		—	NS4	143-601	142	530	152	18.37			
**10**	790	47.22	NS3	17-733	16	60	238	25.92	**5′-GUAUAG**G….	….C**CAAAGGAUAC-3′**	KC853051
		—	NS3a	407-733	406	60	108	11.848			

Pol = RNA polymerase; OC = outer capsid protein; Cap = capping enzyme (guanylyltransferase); Hel = helicase enzyme; T2 = protein with T = 2 symmetry; T13 = Protein with T = 13 symmetry; ViP = viral inclusion body matrix protein; TuP = tubule protein. OC1 and OC2 refer to the larger and smaller outer capsid proteins respectively.

Collectively the terminal non-coding regions (NCR) represent 4.99% of the CORV-MRM1 genome (Table 2). Comparisons with data from GenBank for other mosquito-borne-orbiviruses (MBOs) showed similar or higher percentages, while the tick-borne orbiviruses (TBOs) and *Culicoides*-borne orbiviruses (CBOs) show similar or lower values (Table 2).

**Table 2:** Full genome sequence database available for recognised species of genus *Orbivirus* and their genome coding assignments.

	CORV			UMAV			PHSV			YUOV			BTV-8w			AHSV-1			EHDV-1e			EEV			EUBV			CHUV			GIV			SCRV		
Protein	size in bp	Segment	protein size in aa	size in bp	Segment	protein size in aa	size in bp	Segment	protein size in aa	size in bp	Segment	protein size in aa	size in bp	Segment	protein size in aa	size in bp	Segment	protein size in aa	size in bp	Segment	protein size in aa	size in bp	Segment	protein size in aa	size in bp	Segment	protein size in aa	size in bp	Segment	protein size in aa	size in bp	Segment	protein size in aa	size in bp	Segment	protein size in aa
**Pol**	3925	S1	1290	3933	S1	1299	3987	S1	1311	3993	S1	1315	3944	S1	1302	3966	S1	1305	3942	S1	1302	3963	S1	1307	3962	S1	1307	3930	S1	1295	3897	S1	1285	4089	S1	1345
**T2**	2284	S2	755	2791	S2	905	2856	S2	925	2900	S2	940	2772	S3	901	2792	S3	905	2768	S3	899	3178	S3	902	2773	S3	900	2774	S3	904	2794	S2	908	2747	S2	890
**OC1**	2952	S3	953	2411	S3	797	2747	S3	881	2688	S3	873	2939	S2	961	3218	S2	1056	2968	S2	971	2775	S2	1042	2958	S2	968	3055	S2	1002	1722	S4	551	2024	S4	654
**Cap**	2032	S4	643	2021	S5	648	1996	S4	646	1993	S4	645	1981	S4	644	1978	S4	642	1983	S4	644	1986	S4	646	1984	S4	644	1967	S4	640	1936	S3	635	2017	S3	643
**Tup**	1936	S5	589	2047	S4	580	1784	S5	554	1957	S5	574	1776	S5	552	1748	S5	548	1806	S5	551	1749	S5	548	1763	S5	556	1764	S5	545	1731	S5	531	1657	S5	517
**OC2**	1683	S6	527	1681	S6	529	1695	S6	529	1683	S6	535	1637	S6	526	1564	S6	505	1640	S6	527	1607	S6	515	1658	S6	529	1610	S6	521	1666	S6	537	1664	S6	517
**T13**	1176	S8	354	1171	S8	351	1180	S8	353	1191	S8	355	1156	S7	349	1167	S8	349	1162	S8	349	1175	S8	350	1175	S7	350	1151	S7	348	1181	S7	357	1256	S7	379
**ViP**	1187	S7	371	1357	S7	407	1613	S7	435	1504	S7	435	1125	S8	354	1166	S9	365	1186	S7	373	1181	S7	362	1128	S8	351	1059	S8	333	1172	S8	359	1463	S8	462
**Hel**	1128	S9	344	1103	S9	341	1071	S9	334	1082	S9	338	1049	S9	329	1169	S7	369	1143	S9	360	1080	S9	341	1047	S9	328	877	S9	272	1056	S9	321	764	S9	232
**NS4**	—	S9	152	—	S9	149	—	S9	111	—	S9	113	—	S9	77	—	S7	117	—	S9	76	—	S9	145	—	S9	84	—	S9	83	—	S9	190	—	S9	93
**NS3**	790	S10	238	887	S10	264	819	S10	255	825	S10	253	822	S10	229	763	S10	218	810	S10	228	759	S10	240	856	S10	242	728	S10	211	703	S10	171	764	S10	224
**Total Genome (bp)**	19093			19402			19748			19816			19201			19531			19408			19453			19304			18915			17858			18445		
**%NCR**	4.99			5.71			5.46			5.18			3.96			3.79			4.09			3.57			4.04			3.71			5			4.64		
**G+C (%)**	45.16			41.55			36.66			41.59			44.98			43.46			43.02			45.86			45			39.89			58.13			51.92		
**Vectors**	**Mosquitoes**												***Culicoides spps***																		**Tick or Tick associated**					

**Pol** = Polymerase, **OC1** = Outer capsid protein 1 (VP2 of BTV), **T2** = Inner core protein (T2 symmetry), **Cap** = Capping enzyme, **Tup** = Tubule forming protein or Tubular protein (NS1), **OC2** = Outer capsid protein 2 (VP5 of BTV), **T13** = Outer core protein (T13 symmetry), **ViP** = Viral inclusion body protein (NS2), **Hel** = Helicase protein. NCR and G+C content were calculated for full genome sequences.

Like other orbiviruses, most genome segments of CORV-MRM1 (AUS1960/01), have shorter 5′ than 3′ NCRs, except for Seg-6 (encoding the smaller outer capsid protein VP5(OC2)) which has a longer 5′ NCR and Seg-9 (encoding VP6) which has 3′ and 5′ NCRs of equal length (Table 1). Exceptions also occur in Umatilla virus (UMAV) and Great Island virus (GIV) although the significance of these variations is unclear.

Seg-5 (1,936 bp) of CORV-MRM1 which encodes the ‘tubule’ protein NS1(TuP) (one of the most abundantly expressed orbivirus proteins) is longer than the homologous Seg-5 of the CBOs or TBOs (Table 2) with a long (150 bp) 3′ NCR, but is smaller than that of some other MBOs [1]. Long 3′ NCRs were also observed in the NS1 genome segment of some other MBOs (e.g. UMAV-278 bp; Yunnan orbivirus [YUOV]-205 bp), but not in all other orbiviruses (<118 bp).

The ‘highly conserved’ subcore-shell VP2(T2) protein and the ‘highly variable’ outer-capsid/cell-attachment protein VP3(OC1) of CORV-MRM1, are encoded by Seg-2 and Seg-3, respectively. A similar coding pattern is seen in other MBOs, but the presence of a larger OC1 in the CBOs (including bluetongue virus, the orbivirus ‘type’ species) results in a reversed coding assignment for these two genome-segments (Figure 1 and Table 2).

The size of the highly conserved T13 core-surface protein (VP7 of BTV) is also relatively consistent in most orbiviruses, while the viral inclusion-body matrix-protein, (non-structural protein 2 [NS2(ViP)]) is more variable in size (Table 2). As a result, NS2 of CORV-MRM1 (AUS1960/01) is encoded by Seg-7, while Seg-8 encodes the core-surface protein VP7(T13) (Table 1), in a manner similar to the other MBOs (Table 2). However, this coding-assignment is again reversed in BTV and in some CBOs (EUBV and CHUV), due to variability in the size of NS2 between different viruses [34], [35]. The capping enzyme ‘VP4(CaP)’ of CORV-MRM1 (at 643aa) is smaller than that of the other MBOs and some of the CBOs (Table 2). However, it is encoded by the largest Seg-4 (at 2,032 bp) so far identified in any orbivirus.

Most of the genome segments of CORV-MRM1 (AUS1960/01) except Seg-9 and Seg-10 are monocistronic, encoding a protein from a single large ORF, starting from an initiation codon with a strong Kozak sequence (RNN**AUG**G) [36]. However, like BTV, CORV Seg-10 has two in-frame AUG initiation sites encoding the NS3 and NS3a proteins of 238 aa and 108 aa (starting at 17 bp and 407 bp) respectively (Table 1). The first of these (coding for NS3) has a ‘weak Kozak context’ (GUA**AUG**U), possibly enhancing read-through and initiation of translation from the second ‘in frame’ initiation site (at 407 bp). This has a strong Kozak context (**G**UU**AUGG**), but would express the smallest NS3a in any of the orbiviruses characterized to date.

The first start codon of CORV-MRM1 (AUS1960/01) Seg-9 also has a moderate Kozak sequence (UUG**AUGA**) and a second down-stream ORF. However, this is in the +2 reading-frame (at 143–598 bp), encoding the 152 aa NS4 protein. NS4 has previously been identified in several other orbiviruses and has been characterised in BTV and GIV [24], [37], [38]. The downstream Seg-9 ORF of CORV has a strong Kozak context (**A**GG**AUGG**) and is expected to produce a protein in infected cells. Weak or moderate Kozak sequences have also been observed in several of the genome segments of other orbiviruses, but they still appear to be translated effectively [24], [39].

The G+C content of the CORV genome is 45.16%, which is considerably higher than that of other MBOs [Peruvian horse sickness virus (PHSV) and YUOV with 36.66% and 41.59% respectively] but within the overall G+C range of the insect-borne orbiviruses [36.66% in PHSV (mosquito), to 45.86% in Equine encephalosis virus (EEV) (*Culicoides*)]. However, it is lower than that of the tick-borne or tick-associated orbiviruses [57.29% in GIV and 51.93% in *St Croix River virus* (SCRV)].

### Phylogenetic comparisons of subcore-shell ‘T2’ proteins

**Figure 2:**
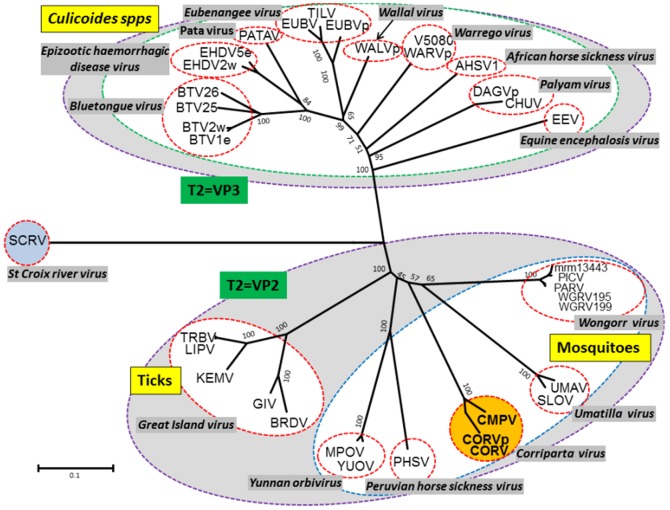
Unrooted neighbour-joining tree for orbivirus subcore-shell T2 proteins. A phylogenetic tree was constructed using distance matrices, generated using the p-distance determination algorithm and pairwise deletion parameters in MEGA 5 (1000 bootstrap replicates) [65]. Since many of the available sequences are incomplete, the analysis is based on partial sequences (aa 393 to 548, numbered with reference to the aa sequence of BTV-VP3(T2)). The numbers at nodes indicate bootstrap confidence values after 1000 replications. The tree shown in Figures 3 and 4 were drawn using the same parameters. The CORV and CMPV isolates are shown in red font in the amber coloured circle. Full names of virus isolates and accession numbers of T2 protein sequences used for comparative analysis are listed in Table S1 (supplementary data). ‘e’ and ‘w’ after serotype number indicate eastern and western topotype strains, respectively.

Comparison of homologous orbivirus proteins using BlastX analysis identified VP2, encoded by Seg-2 of CORV, as the sub-core shell ‘T2’ protein (equivalent to VP3(T2) of BTV and VP2(T2) of YUOV and SCRV). An unrooted neighbour-joining (NJ) phylogenetic tree (Figure 2) constructed for different orbivirus T2 protein sequences (listed in Table S1 supplementary data) identified two major clusters (Figure 2). The larger group in which VP3(T2) is encoded by Seg-3, comprises the *Culicoides* transmitted orbiviruses (including BTV, AHSV, EHDV, WALV, *Eubenangee virus* [EUBV], WARV and *Palyam virus* [PALV]). However, the second group in which VP2(T2) is encoded by Seg-2, includes two sub-groups transmitted by ticks (the Great Island viruses), or by mosquitoes (WGRV, UMAV, PHSV and YUOV) respectively. SCRV, which has previously been suggested as a tick-orbivirus rather than a tick-borne-orbivirus [40], branches separately from these groups and appears to be more distantly related to other orbiviruses. VP2(T2) of CORV-MRM1 (AUS1960/01) clusters very closely with partial sequence data available for CMPV(98.78%/98.99% aa/nt identity Table 3 and 4), within the mosquito-borne group, indicating that they belong to the same virus species. These two viruses show lower levels of identity with the other *Orbivirus* species analysed (<58.53%/57.98% aa/nt identity - Table 3).

**Table 3:** Percent nucleotide (nt) and amino acid (aa) identities of Corriparta virus MRM1(AUS1960/01) with other orbiviruses.

Sl no.	Orbivirus species	Abbreviation	T2		VP1		T13	
			aa	nt	aa	nt	aa	nt
**1**	*Bluetongue virus*	**BTV**	37.11	47.33	47.40	52.41	25.07	38.71
		**BTV25**	36.22	47.00	46.84	52.12	23.63	38.14
		**BTV26**	37.11	46.56	46.84	51.68	24.21	37.85
**2**	*African horse sickness virus*	**AHSV**	37.06	47.64	47.90	53.03	23.85	39.85
**3**	*Epizootic haemorrhagic disease virus*	**EHDV**	37.19	46.88	47.40	53.06	24.50	39.67
**4**	*Eubenangee virus*	**EUBV**	36.60	47.53	47.60	52.88	27.30	42.53
**5**	*Palyam virus*	**PALV**	35.77	47.14	50.51	53.99	27.67	41.02
**6**	*Equine encephalosis virus*	**EEV**	33.96	45.36	46.61	51.26	24.71	39.18
**7**	*Warrego virus*	**WARV**	44.72	51.78	—	—	—	—
**8**	*Wlallal virus*	**WALV**	44.64	50.83	—	—	—	—
**9**	*Umatilla virus*	**UMAV**	50.17	55.21	59.66	58.12	42.57	47.62
		**SLOV**	50.39	54.29	57.94	57.23		
**10**	*Peruvian horse sickness virus*	**PHSV**	46.37	53.45	52.06	55.38	33.52	43.94
**11**	*Yunnan orbivirus*	**YUOV**	44.78	53.16	51.44	54.90	34.09	45.36
**12**	*Corriparta virus* *	**CORV**	98.78	98.99	—	—	—	—
**13**	*Wongorr virus*	**WGRV**	58.53	57.98	—	—	—	—
**14**	*Great Island virus*	**GIV**	45.20	49.87	50.51	52.13	32.95	41.57
**15**	*St Croix river virus*	**SCRV**	24.15	38.46	39.01	45.23	22.03	35.94
**16**	Pata virus	**PATAV**	36.19	46.84	47.23	52.28	26.59	40.75

* data previously published for corriparta virus MRM1 (Ac No. AF530086).

**Table 4:** Percent identities between Corriparta virus (CORV-MRM1) (AUS1960/01) and California mosquito pool virus (CMPV) proteins and genome segments.

Segment (Protein)	Seg-1/VP1 (Pol)	Seg-2/VP2 (T2)	Seg-3/VP3 (OC1)	Seg-4/VP4 (CaP)	Seg-5/NS1 (TuP)	Seg-6/VP5 (OC2)	Seg-7/NS2 (ViP)	Seg-8/VP7 (T13)	Seg-9#/VP6 (Hel) & NS4	Seg-10/(NS3)
**Genome segment of CORV (bp)**	3,925	2,952	2,284	2,032	1,936	1,683	1,187	1,176	1,128	790
**Partial sequence of CMPV (bp)** *	—	358	—	288	—	646	—	737	596	—
**% aa identity (CORV vs CMPV)**	—	91.6	—	75	—	86.2	—	91.7	42.1/55.9§	—
**% nt identity (CORV vs CMPV)**	—	75.9	—	67.4	—	74.3	—	77.6	61.6/63.2§	—

* Only partial CMPV sequences are available in GenBank.

# Identities calculated using reverse complement sequences of CMPV.

§ Indicates identities of NS4.

### Phylogenetic comparisons of VP1(Pol) proteins

**Figure 3:**
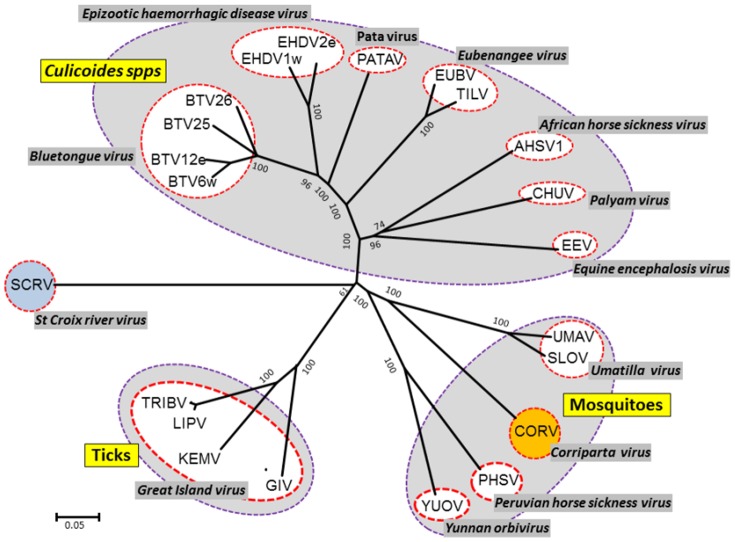
Unrooted neighbour-joining for orbivirus polymerase VP1(Pol) proteins. An unrooted NJ phylogenetic tree for orbivirus VP1(Pol) proteins was constructed using a p-distance algorithm and pairwise deletion parameters, as indicated in Figure 1. The CORV-MRM1 isolate characterised in this study is indicated in red font in amber coloured circle. Full names of virus isolates and accession numbers of polymerase sequences used for comparative analysis are listed in Table S1 (supplementary data). ‘e’ and ‘w’ after serotype number indicate eastern and western topotype strains, respectively.

Seg-1, which encodes the viral RNA polymerase VP1(Pol), is highly conserved across the genus *Orbivirus*, and provides a target for sequencing and RT-PCR assays to identify virus isolates at both species and genus levels [1], [24], [31], [40]. The aa sequence of VP1(Pol) from CORV-MRM1 (AUS1960/01) was compared with representatives of other *Orbivirus* species to construct an unrooted NJ phylogenetic tree (Figure 3). Three major clusters were identified, corresponding to the different vector-groups: CBOs, MBOs and TBOs. CORV-MRM1 (AUS1960/01) again clusters as a distinct species, close to other MBOs (>51.44/54.9% aa/nt identity - Table 3), with more distant relationships to VP1 of the *Culicoides* and tick-borne groups (<50.51%/53.99% aa/nt identity - Table 3).

### Phylogenetic comparisons of outer-core ‘T13’ proteins

**Figure 4:**
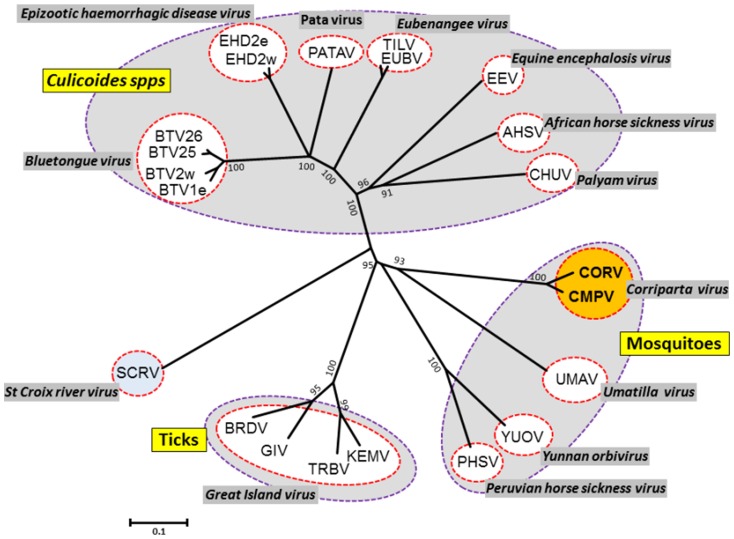
Unrooted neighbour-joining for orbivirus outer-core VP7(T13) proteins. An unrooted NJ phylogenetic tree for orbivirus VP7(T13) proteins was constructed using a p-distance algorithm and pairwise deletion parameters, as indicated in Figure 1. The CORV-MRM1 and CMPV isolates are shown in red font in amber coloured circle. Full names of virus isolates and accession numbers of T13 protein sequences used for comparative analysis are listed in Table S1 (supplementary data). ‘e’ and ‘w’ after serotype number indicate eastern and western strains, respectively.

The orbivirus core-surface protein VP7(T13) is immunodominant and represents a major serogroup-specific antigen [21]. An unrooted phylogenetic tree for VP7(T13) (encoded by Seg-8 of CORV-MRM1) (Figure 4) shows a similar topology to the VP2(T2) and VP1(Pol) trees (Figures 2 and 3), grouping viruses according to their vectors. CORV-MRM1 clusters with CMPV (sharing 91.7%/77.6% aa/nt identity) as members of a distinct species (Table 4), showing lower identities with the other MBOs (<42.57%/47.62% aa/nt identities (with UMAV)), or the CBOs and TBOs (<32.95%/41.57% aa/nt identities (with GIV)) (Table 3).

### Relationships between CORV and CMPV

The sequences of CORV-MRM1 Seg-4/VP4(Cap), Seg-6/VP5(OC2), and Seg-9/VP6(Hel)/NS4 were also compared with the incomplete sequence data available for CMPV (Table 4). CMPV again grouped closely with CORV-MRM1 (AUS1960/01), sharing aa/nt identities of 75/67.4% and 86.2/74.3% in VP4(Cap) and VP5(OC2) respectively, further supporting the identification of CMPV as a member of *Corriparta virus* species. Seg-9 of CORV-MRM1 (coding for NS4 and VP6(Hel)) shares only 42.6/61.6% aa/nt identities with CMPV. However, no closer matches were found in either of these proteins with members of other *Orbivirus* species. Further analysis of CMPV Seg-9 confirmed the presence of an alternate ORF, encoding a 153 aa NS4 protein, which shares 55.9%/63.2% aa/nt identity with NS4 of CORV-MRM1.

## Discussion

Collectively the orbiviruses infect a wide range of hosts and are transmitted by a diverse group of vectors, including *Culicoides*, mosquitoes, sand flies and ticks [7]. Initially, the different ‘serogroups’ of orbiviruses, which are now recognised as distinct virus species, were identified by CF, AGID, immunofluourescence (IF) tests and/or enzyme-linked immunosorbent assays (ELISA). However, low level serological cross-reactions have been detected between some of the more closely related *Orbivirus* species (for example between isolates of BTV (the *Orbivirus* type species) and EHDV [41] making it difficult to conclusively identify the species of new isolates by these techniques alone. The high quality reference-strains and antisera needed for these assays are not widely available for all of the existing serogroups/species, and may themselves represent a biosecurity risk. In addition, serological methods do not generate absolute quantitative values for the relatedness of individual virus strains.

In contrast, nucleotide sequence data for reference orbivirus strains and novel isolates can be compared and transmitted easily between laboratories, without risk, providing highly reproducible and fully quantitative numerical values for the relatedness of each genome segment/protein. These data can also be used to unambiguously identify different genome segments, proteins, virus species, topotypes and serotypes [1], [25], [33], [39], [42].

Due to their economic significance, full genomes of multiple isolates (including reference strains) of BTV, EHDV and AHSV have already been characterized [23], [43]–[49]. These data have supported development of rapid and reliable molecular methods and diagnostic tools (RT-PCR assays) for identification of virus serogroup/species and serotype [27]–[31], [49]–[51].

Sequence variations in the outermost orbivirus capsid and cell-attachment protein, VP2(OC1) of BTV, correlate with both the geographic origin of the virus (topotype) and with its serotype [42], [47]. In contrast sequence variation in the core proteins VP1(Pol) and T2 (VP3 of BTV) correlate only with virus genus, species and topotype [1], [32], [33], [39], [40]. However, a lack of full-genome sequence data for all 22 recognized *Orbivirus* species has hindered identification of isolates belonging to novel *Orbivirus* species and development of nucleic acid based diagnostic tests.

The full-genome sequence data presented here for CORV-MRM1 (AUS1960/01) provides a primary reference for identification of other (novel) members of the species *Corriparta virus* (CORV). Conserved nucleotide sequences are present at both the upstream and downstream termini of the genome segments of different *Orbivirus* species [2], [52]. All of the genome segments of CORV-MRM1 (AUS1960/01) contain the terminal sequences (5′-GUAUAG…..CAAAGGAUAC-3′) showing several differences from those of BTV (e.g. 5′-GUUAAA……..ACUUAC-3′). The significance of a longer conserved 3′ terminal region in CORV-MRM1 is unknown, although it has been suggested that these regions may play a role in initiation of transcription or translation of the RNA or its packaging during virus replication [53], [54].

The orbivirus proteins VP1(Pol), ‘T2’ and ‘T13’, which are highly conserved have taken priority in development of molecular diagnostic assays and in phylogenetic analyses [24], [29], [31], [32], [55]. Studies with large numbers of different BTV and EHDV isolates show >73%, >83% and >73% intra-species aa identities in VP1, T2 and T13 respectively, providing useful markers for the identification and classification of existing and novel orbivirus isolates [1], [40], [44].

CORV-MRM1 shares less than 60% aa identity (Table 3) in VP1, T2 and T13, with members of the other recognised *Orbivirus* species, confirming the classification of *Corriparta virus* as a distinct species. CORV-MRM1 is most closely related to other MBO species, particularly *Umatilla virus* (UMAV), with 59.66% and 50.17% aa identity in VP1 and T2 proteins respectively (Table 3). However, CORV-MRM1 shows 91.6%/75.9% aa/nt identity and 91.7%/77.6% aa/nt identity to CMPV in its T2 and T13 protein/gene sequences, indicating that they belong to the same virus species and therefore that CMPV does not represent a new species as previously suggested [5].

Phylogenetic analyses show that although the size of the orbivirus sub-core-shell T2 protein is relatively constant across the genus (between 890 aa in SCRV to 953 aa in CORV), the size of the larger of the two outer coat proteins (OC1) is much more variable (between 551 aa in GIV to 1056 aa in AHSV). This results in a change in relative size, order and numbering of the genome segments encoding the T2 and OC1 proteins, between the tick-associated/transmitted orbiviruses [VP2(T2) and VP4(OC1) - encoded by Seg-2 and Seg-4], the MBOs [VP2(T2) and OC1(VP3) - encoded by Seg-2 and Seg-3 respectively], and the CBOs [VP3(T2) and VP2(OC1) - encoded Seg-3 and Seg-2 respectively] [1], [23], [34], [39], [40], [56].

Previous studies have suggested that the MBOs have evolved from tick-borne ancestors, with CBOs being last to evolve [39]. The concatermerisation of orbivirus genome segments and subsequent mutations may provide a mechanism that can progressively increase the size of individual genome segments [39], [56]. It may therefore be significant that the size of OC1 increases in the order: TBOs (551 aa in GIV and 654 aa in SCRV), MBOs (755 aa in CORV to 881 aa in PHSV) and CBOs (961 aa in BTV to 1056 aa in AHSV-1), (Table 2).

Previous studies also indicate that the orbiviruses have evolved through a process of ‘co-speciation’ with their vectors [39]. Phylogenetic analyses of the conserved Pol, T2 and T13 proteins (presented here - Fig. 2, 3 and 4), show consistent grouping of the CBOs, MBOs and TBOs. In each case CORV-MRM1 groups with the other MBOs (WGRV, UMAV, PHSV and YUOV).

Corriparta viruses have been isolated in Australia, Africa and South America [11]. However, the data presented here clearly identify CMPV, which was isolated in North America [5], as a member of the species *Corriparta virus*. The occurrence of these closely related viruses in the Americas and Australia indicates that there has been spread of viruses between these regions, which could be due to movement of infected hosts or vectors. Similar movements are also suggested by the detection of other orbiviruses (UMAV and PHSV, and individual serotypes of BTV and EHDV) in more than one continent, e,g, in Australia, Africa and the Americas [1], [28], [57]–[59]. Additional strains of each serogroup/species, from different locations/origins, need to be isolated and characterised/sequenced to better understand their geographical distribution and its significance.

The sequences and relative sizes of the VP1(Pol), T2 and OC1 proteins are important evolutionary markers that can help differentiate/group orbiviruses by species, serotypes and topotype. The sequence data generated in this study will facilitate the use of phylogenetic analyses to identify other novel isolates belonging to the *Corriparta virus* species, as well as helping to identify the arthropod vectors involved in their transmission. Further studies are still needed to define the different serotype and topotypes of CORV.

## Materials and Methods

### Virus propagation

CORV-MRM1 (AUS1960/01), obtained at passage level MB6/BHK2 from the Orbivirus Reference Collection at The Pirbright Institute, was propagated in BHK-21 cell monolayers [clone 13 obtained from European Collection of Animal cell Cultures (ECACC – 84100501)], in Dulbecco's minimum essential medium (DMEM) supplemented with antibiotics (100 units/ml penicillin and 100 µg/ml streptomycin) and 2 mM glutamine. Infected cell-cultures were incubated at 37°C until they showed widespread (100%) cytopathic effects (CPE). Then viruses were harvested, aliquoted and used for the extraction of viral dsRNA. All virus isolates used in these studies were obtained from diagnostic samples of naturally infected animals and were taken as a part of normal diagnostic investigations by qualified veterinarians in the individual countries.

### Extraction and purification of CORV dsRNA

Cell monolayers showing 100% CPE after infection with CORV-MRM1, were harvested and pelleted at 3000 *g* for 5 min. The viral dsRNA was released and purified using TRIzol® reagent (Invitrogen) as described by Attoui *et al* [60]. Briefly, the infected cell pellet was lysed in 1 ml of TRIzol®, then 0.2 volume of choloroform was added, vortexed and the mixture incubated on ice for 10 min. The aqueous phase, containing total RNA, was separated from the phenol-chloroform phase by centrifugation at 10,000 *g* for 10 min before 900 µl of isopropanol was added prior to incubation at −20°C for 2 hours. The RNA was pelleted at 10,000 *g* for 10 min, washed with 70% ethanol, air dried and dissolved in 100 µl of nuclease free water (NFW). Single stranded RNA (ssRNA) was removed by precipitation with 2M LiCl (Sigma) at 4°C overnight, followed by centrifugation at 10,000 *g* for 5 min. An equal volume of isopropanol, containing 750 mM ammonium acetate, was mixed with the supernatant. After precipitation at −20°C for 2 hours, the viral dsRNA was pelleted at 10,000 *g* for 10 min, washed with 70% ethanol, air dried and suspended in 50 µl of NFW. The RNA was either used immediately or stored at −20°C.

### Reverse transcription and PCR amplification

CORV-MRM1 genome segments were reverse-transcribed into cDNA using the full-length amplification (FLAC) technique described by Maan *et al.* [61]. Briefly, a 35 base oligonucleotide ‘anchor-primer’, with a phosphorylated 5′ terminus, was ligated to the 3′ ends of the viral dsRNAs using the T4 RNA ligase overnight at 16°C. Then dsRNA segments were fractionated on 1% agarose gel and recovered from the gel using a ‘silica binding’ method (RNaid® kit, MP Biomedicals) as per the manufacturer's instructions. The dsRNA eluted in NFW, was denatured at 99°C for 5 minutes, and then snap chilled on ice before synthesising first-strand cDNA using RT system (Promega). The resulting cDNAs were amplified using primers complementary to the anchor primer and high fidelity KOD polymerase enzyme (Novagen). PCR amplicons were analyzed by agarose gel electrophoresis.

### Cloning and sequencing of cDNA segments

cDNA amplicons were purified and cloned into the ‘pCR®-Blunt’ vector supplied with the Zero Blunt® PCR Cloning Kit (Invitrogen). Recombinant plasmid-vectors containing CORV-MRM1 inserts were transformed into One Shot® TOP10 competent cells supplied with the cloning kit. Clones containing the desired inserts were identified by colony touch PCR using M13 universal primers. Plasmids were extracted from the clones identified, using the QIAprep Spin MiniPrep Kit (Qiagen). The plasmids and PCR products were sequenced using an automated ABI 3730 DNA sequencer (Applied Biosystems).

### Sequence and phylogenetic analysis

‘Raw’ ABI sequence data was assembled into ‘contigs’ using the SeqManII sequence analysis package (DNAstar version 5.0). The ORFs of CORV-MRM1 genome segments were identified and translated to aa sequences for further analysis using EditSeq (DNAstar version 5.0). The putative function of each protein was identified by Blast X comparisons to homologous orbivirus (BTV) proteins in GenBank (http://blast.ncbi.nlm.nih.gov/Blast.cgi?CMD=Web&PAGE_TYPE=BlastHome). Multiple alignments of consensus sequences were performed using Clustal X (Version 2.0) [62], Clustal Omega (http://www.ebi.ac.uk/Tools/msa/clustalo/) and MAFFT [63] to ensure proper alignment. Aligned protein sequences were back translated to nucleotide sequences using DAMBE [64]) or RevTrans 1.4 server available online (http://www.cbs.dtu.dk/services/RevTrans/) for further nucleotide analysis. Pairwise distance (aa and nt) calculations and phylogenetic trees constructions were done using MEGA 5 software [65] with the p-distance parameter and neighbour-joining method [66]. GenBank nucleotide accession numbers of polymerase (VP1), T2 and T13 protein sequences that were used in phylogenetic analyses are provided in Table S1 (supplementary data).

## Supporting Information

Table S1 **Nucleotide accession numbers for sequences used in phylogenetic analysis.**
(DOCX)

## References

[1] Belaganahalli MN, Maan S, Maan NS, Tesh R, Attoui H, et al (2011) Umatilla virus genome sequencing and phylogenetic analysis: identification of stretch lagoon orbivirus as a new member of the Umatilla virus species. PLoS One 6: e23605. 21897849 10.1371/journal.pone.0023605 PMC3163642

[2] Mertens PPC, Maan S, Samuel A, Attoui H (2005) Orbiviruses, Reoviridae. In: Fauquet CM, M.A. Mayo, J. Maniloff, U. Desselberger and L.A. Ball, editor. Virus Taxonomy Eighth Report of the International Committee on Taxonomy of Viruses. London: Elsevier/Academic Press. pp. pp. 466–483.

[3] Martins LC, Diniz JA, Silva EV, Barros VL, Monteiro HA, et al (2007) Characterization of Minacu virus (Reoviridae: Orbivirus) and pathological changes in experimentally infected newborn mice. Int J Exp Pathol 88: 63–73. 17244340 10.1111/j.1365-2613.2006.00516.x PMC2517288

[4] Brown SE, Gorman BM, Tesh RB, Knudson DL (1992) Isolation of bluetongue and epizootic hemorrhagic disease viruses from mosquitoes collected in Indonesia. Vet Microbiol 32: 241–252. 1333672 10.1016/0378-1135(92)90147-l

[5] Victoria JG, Kapoor A, Dupuis K, Schnurr DP, Delwart EL (2008) Rapid identification of known and new RNA viruses from animal tissues. PLoS Pathog 4: e1000163. 18818738 10.1371/journal.ppat.1000163 PMC2533695

[6] Kapoor A, Tesh RB, Duraisamy R, Popov VL, Travassos da Rosa AP, et al (2013) A novel mosquito-borne Orbivirus species found in South-east Asia. J Gen Virol 94: 1051–1057. 23364187 10.1099/vir.0.046748-0 PMC3998237

[7] Attoui H, Mertens PPC, Becnel J, Belaganahalli M, Bergoin M, et al., editors (2012) Reoviridae. London, UK: Elsevier Academic Press. 497–650 p.

[8] Karabatsos N (1985) International catalogue of arboviruses including certain other viruses of vertebrates. San Antonio: American Society of Tropical Medicine and Hygiene.

[9] Attoui H, Maan SS, Anthony SJ, Mertens PPC (2009) Bluetongue virus, other orbiviruses and other reoviruses: Their relationships and taxonomy. In: Mellor PS, Baylis M, Mertens PPC, editors. Bluetongue monograph. 1 ed. London: Elsevier/Academic Press. pp. 23–552.

[10] Carley JG, Standfast HA (1969) Corriparta virus: properties and multiplication in experimentally-inoculated mosquitoes. Am J Epidemiol 89: 583–592. 4238947 10.1093/oxfordjournals.aje.a120971

[11] Gonzalez HA, Knudson DL (1987) Genetic relatedness of corriparta serogroup viruses. J Gen Virol 68 (Pt 3) 661–672. 3819699 10.1099/0022-1317-68-3-661

[12] Boughton CR, Hawkes RA, Naim HM (1990) Arbovirus infection in humans in NSW: seroprevalence and pathogenicity of certain Australian bunyaviruses. Aust N Z J Med 20: 51–55. 2108660 10.1111/j.1445-5994.1990.tb00371.x

[13] Whitehead RH, Doderty RL, Domrow R, Standfast HA, Wetters EJ (1968) Studies of the epidemiology of arthropod-borne virus infections at Mitchell River Mission, Cape York Peninsula, North Queensland. 3. Virus studies of wild birns, 1964–1967. Trans R Soc Trop Med Hyg 62: 439–445. 5659236 10.1016/0035-9203(68)90096-5

[14] Doherty RL, Whitehead RH, Wetters EJ, Gorman BM, Carley JG (1970) A survey of antibody to 10 arboviruses (Koongol group, Mapputta group and ungrouped) isolated in Queensland. Trans R Soc Trop Med Hyg 64: 748–753. 5533929 10.1016/0035-9203(70)90017-9

[15] Doherty RL, Carley JG, Mackerras MJ, Marks EN (1963) Studies of arthropod-borne virus infections in Queensland. III. Isolation and characterization of virus strains from wild-caught mosquitoes in North Queensland. Aust J Exp Biol Med Sci 41: 17–39. 14028387 10.1038/icb.1963.2

[16] Liehne PF, Anderson S, Stanley NF, Liehne CG, Wright AE, et al (1981) Isolation of Murray Valley encephalitis virus and other arboviruses in the Ord River Valley 1972–1976. Aust J Exp Biol Med Sci 59: 347–356. 6117274 10.1038/icb.1981.29

[17] van Regenmortel MH, Mahy BW (2004) Emerging issues in virus taxonomy. Emerg Infect Dis 10: 8–13. 15078590 10.3201/eid1001.030279 PMC3322749

[18] Brown SE, Gonzalez HA, Bodkin DK, Tesh RB, Knudson DL (1988) Intra- and inter-serogroup genetic relatedness of orbiviruses. II. Blot hybridization and reassortment in vitro of epizootic haemorrhagic disease serogroup, bluetongue type 10 and Pata viruses. J Gen Virol 69 (Pt 1) 135–147. 2826662 10.1099/0022-1317-69-1-135

[19] Walker PJ, Taylor J, Gorman BM (1989) Detection of reassortant orbiviruses (Wallal serogroup) in a prototype strain isolated from a pool of biting midges (Culicoides dycei). J Gen Virol 70 (Pt 4) 1011–1016. 2732705 10.1099/0022-1317-70-4-1011

[20] Walker PJ, Mansbridge JN, Gorman BM (1980) Genetic Analysis of Orbiviruses by Using RNase T(1) Oligonucleotide Fingerprints. J Virol 34: 583–591. 16789197 10.1128/jvi.34.3.583-591.1980 PMC288745

[21] Gumm ID, Newman JF (1982) The preparation of purified bluetongue virus group antigen for use as a diagnostic reagent. Arch Virol 72: 83–93. 6285867 10.1007/BF01314453

[22] Standfast HA, Dyce AL, St George TD, Muller MJ, Doherty RL, et al (1984) Isolation of arboviruses from insects collected at Beatrice Hill, Northern Territory of Australia, 1974–1976. Aust J Biol Sci 37: 351–366. 6152599 10.1071/bi9840351

[23] Maan S, Maan NS, Ross-smith N, Batten CA, Shaw AE, et al (2008) Sequence analysis of bluetongue virus serotype 8 from the Netherlands 2006 and comparison to other European strains. Virology 377: 308–318. 18570969 10.1016/j.virol.2008.04.028

[24] Belaganahalli MN, Maan S, Maan NS, Nomikou K, Pritchard I, et al (2012) Full Genome Sequencing and Genetic Characterization of Eubenangee Viruses Identify Pata Virus as a Distinct Species within the Genus Orbivirus. PLoS One 7: e31911. 22438872 10.1371/journal.pone.0031911 PMC3305294

[25] Maan S, Maan NS, Nomikou K, Batten C, Antony F, et al (2011) Novel bluetongue virus serotype from Kuwait. Emerg Infect Dis 17: 886–889. 21529403 10.3201/eid1705.101742 PMC3321788

[26] Attoui H, Mohd Jaafar F, Belhouchet M, Aldrovandi N, Tao S, et al (2005) Yunnan orbivirus, a new orbivirus species isolated from Culex tritaeniorhynchus mosquitoes in China. J Gen Virol 86: 3409–3417. 16298988 10.1099/vir.0.81258-0

[27] Maan NS, Maan S, Nomikou K, Belaganahalli MN, Bachanek-Bankowska K, et al (2011) Serotype specific primers and gel-based rt-PCR assays for ‘typing’ african horse sickness virus: identification of strains from Africa. PLoS One 6: e25686. 22028787 10.1371/journal.pone.0025686 PMC3197586

[28] Maan NS, Maan S, Nomikou K, Johnson DJ, El Harrak M, et al (2010) RT-PCR assays for seven serotypes of epizootic haemorrhagic disease virus & their use to type strains from the Mediterranean region and North America. PLoS One 5: e12782. 20862243 10.1371/journal.pone.0012782 PMC2941451

[29] Mertens PP, Maan NS, Prasad G, Samuel AR, Shaw AE, et al (2007) Design of primers and use of RT-PCR assays for typing European bluetongue virus isolates: differentiation of field and vaccine strains. J Gen Virol 88: 2811–2823. 17872535 10.1099/vir.0.83023-0

[30] Maan NS, Maan S, Belaganahalli MN, Ostlund EN, Johnson DJ, et al (2012) Identification and Differentiation of the Twenty Six Bluetongue Virus Serotypes by RT–PCR Amplification of the Serotype-Specific Genome Segment 2. PLoS One 7: e32601. 22389711 10.1371/journal.pone.0032601 PMC3289656

[31] Shaw AE, Monaghan P, Alpar HO, Anthony S, Darpel KE, et al (2007) Development and initial evaluation of a real-time RT-PCR assay to detect bluetongue virus genome segment 1. J Virol Methods 145: 115–126. 17586061 10.1016/j.jviromet.2007.05.014

[32] Pritchard LI, Gould AR, Wilson WC, Thompson L, Mertens PP, et al (1995) Complete nucleotide sequence of RNA segment 3 of bluetongue virus serotype 2 (Ona-A). Phylogenetic analyses reveal the probable origin and relationship with other orbiviruses. Virus Res 35: 247–261. 7785314 10.1016/0168-1702(94)00072-k

[33] Nomikou K, Dovas CI, Maan S, Anthony SJ, Samuel AR, et al (2009) Evolution and phylogenetic analysis of full-length VP3 genes of Eastern Mediterranean bluetongue virus isolates. PLoS One 4: e6437. 19649272 10.1371/journal.pone.0006437 PMC2713410

[34] Mertens PP, Brown F, Sangar DV (1984) Assignment of the genome segments of bluetongue virus type 1 to the proteins which they encode. Virology 135: 207–217. 6328750 10.1016/0042-6822(84)90131-4

[35] Pedley S, Mohamed ME, Mertens PP (1988) Analysis of genome segments from six different isolates of bluetongue virus using RNA-RNA hybridisation: a generalised coding assignment for bluetongue viruses. Virus Res 10: 381–390. 2842980 10.1016/0168-1702(88)90078-0

[36] Kozak M (1981) Possible role of flanking nucleotides in recognition of the AUG initiator codon by eukaryotic ribosomes. Nucleic Acids Res 9: 5233–5252. 7301588 10.1093/nar/9.20.5233 PMC327517

[37] Belhouchet M, Mohd Jaafar F, Firth AE, Grimes JM, Mertens PP, et al (2011) Detection of a fourth orbivirus non-structural protein. PLoS One 6: e25697. 22022432 10.1371/journal.pone.0025697 PMC3192121

[38] Ratinier M, Caporale M, Golder M, Franzoni G, Allan K, et al (2011) Identification and characterization of a novel non-structural protein of bluetongue virus. PLoS Pathog 7: e1002477. 22241985 10.1371/journal.ppat.1002477 PMC3248566

[39] Belhouchet M, Mohd Jaafar F, Tesh R, Grimes J, Maan S, et al (2010) Complete sequence of Great Island virus and comparison with the T2 and outer-capsid proteins of Kemerovo, Lipovnik and Tribec viruses (genus Orbivirus, family Reoviridae). J Gen Virol 91: 2985–2993. 20739272 10.1099/vir.0.024760-0

[40] Attoui H, Stirling JM, Munderloh UG, Billoir F, Brookes SM, et al (2001) Complete sequence characterization of the genome of the St Croix River virus, a new orbivirus isolated from cells of Ixodes scapularis. J Gen Virol 82: 795–804. 11257184 10.1099/0022-1317-82-4-795

[41] Borden EC, Shope RE, Murphy FA (1971) Physicochemical and morphological relationships of some arthropod-borne viruses to bluetongue virus–a new taxonomic group. Physiocochemical and serological studies. J Gen Virol 13: 261–271. 4333714 10.1099/0022-1317-13-2-261

[42] Maan S, Maan NS, Samuel AR, Rao S, Attoui H, et al (2007) Analysis and phylogenetic comparisons of full-length VP2 genes of the 24 bluetongue virus serotypes. J Gen Virol 88: 621–630. 17251581 10.1099/vir.0.82456-0

[43] Maan S, Maan NS, Nomikou K, Veronesi E, Bachanek-Bankowska K, et al (2011) Complete genome characterisation of a novel 26th bluetongue virus serotype from kuwait. PLoS One 6: e26147. 22031822 10.1371/journal.pone.0026147 PMC3198726

[44] Maan S, Maan NS, van Rijn PA, van Gennip RG, Sanders A, et al (2010) Full genome characterisation of bluetongue virus serotype 6 from the Netherlands 2008 and comparison to other field and vaccine strains. PLoS One 5: e10323. 20428242 10.1371/journal.pone.0010323 PMC2859060

[45] Anthony SJ, Maan N, Maan S, Sutton G, Attoui H, et al (2009) Genetic and phylogenetic analysis of the core proteins VP1, VP3, VP4, VP6 and VP7 of epizootic haemorrhagic disease virus (EHDV). Virus Res 145: 187–199. 19632280 10.1016/j.virusres.2009.07.011

[46] Anthony SJ, Maan N, Maan S, Sutton G, Attoui H, et al (2009) Genetic and phylogenetic analysis of the non-structural proteins NS1, NS2 and NS3 of epizootic haemorrhagic disease virus (EHDV). Virus Res 145: 211–219. 19665508 10.1016/j.virusres.2009.07.019

[47] Anthony SJ, Maan S, Maan N, Kgosana L, Bachanek-Bankowska K, et al (2009) Genetic and phylogenetic analysis of the outer-coat proteins VP2 and VP5 of epizootic haemorrhagic disease virus (EHDV): comparison of genetic and serological data to characterise the EHDV serogroup. Virus Res 145: 200–210. 19632281 10.1016/j.virusres.2009.07.012

[48] Potgieter AC, Steele AD, van Dijk AA (2002) Cloning of complete genome sets of six dsRNA viruses using an improved cloning method for large dsRNA genes. J Gen Virol 83: 2215–2223. 12185276 10.1099/0022-1317-83-9-2215

[49] Bankowska K (2013) Genetic studies of African horse sickness virus: A thesis submitted in partial fulfilment for the degree of Doctor of Philosophy. University of Oxford.

[50] Orru G, Ferrando ML, Meloni M, Liciardi M, Savini G, et al (2006) Rapid detection and quantitation of Bluetongue virus (BTV) using a Molecular Beacon fluorescent probe assay. J Virol Methods 137: 34–42. 16876884 10.1016/j.jviromet.2006.05.028

[51] Bremer CW, Viljoen GJ (1998) Detection of African horsesickness virus and discrimination between two equine orbivirus serogroups by reverse transcription polymerase chain reaction. Onderstepoort J Vet Res 65: 1–8. 9629584

[52] Mertens PP, Sangar DV (1985) Analysis of the terminal sequences of the genome segments of four orbiviruses. Virology 140: 55–67. 2981457 10.1016/0042-6822(85)90445-3

[53] Rao CD, Kiuchi A, Roy P (1983) Homologous terminal sequences of the genome double-stranded RNAs of bluetongue virus. J Virol 46: 378–383. 6302309 10.1128/jvi.46.2.378-383.1983 PMC255139

[54] Roner MR, Gaillard RK Jr, Joklik WK (1989) Control of reovirus messenger RNA translation efficiency by the regions upstream of initiation codons. Virology 168: 292–301. 2916327 10.1016/0042-6822(89)90269-9

[55] Sakamoto K, Punyahotra R, Mizukoshi N, Ueda S, Imagawa H, et al (1994) Rapid detection of African horsesickness virus by the reverse transcriptase polymerase chain reaction (RT-PCR) using the amplimer for segment 3 (VP3 gene). Arch Virol 136: 87–97. 8002793 10.1007/BF01538819

[56] Anthony SJ, Darpel KE, Belaganahalli MN, Maan N, Nomikou K, et al (2011) RNA segment 9 exists as a duplex concatemer in an Australian strain of epizootic haemorrhagic disease virus (EHDV): Genetic analysis and evidence for the presence of concatemers as a normal feature of orbivirus replication. Virology 420: 164–171. 21968198 10.1016/j.virol.2011.09.009

[57] Gibbs EP, Calisher CH, Tesh RB, Lazuick JS, Bowen R, et al (1989) Bivens arm virus: a new rhabdovirus isolated from Culicoides insignis in Florida and related to Tibrogargan virus of Australia. Vet Microbiol 19: 141–150. 2650459 10.1016/0378-1135(89)90079-5

[58] Attoui H, Mendez-Lopez MR, Rao S, Hurtado-Alendes A, Lizaraso-Caparo F, et al (2009) Peruvian horse sickness virus and Yunnan orbivirus, isolated from vertebrates and mosquitoes in Peru and Australia. Virology 394: 298–310. 19766284 10.1016/j.virol.2009.08.032

[59] Allison AB, Goekjian VH, Potgieter AC, Wilson WC, Johnson DJ, et al (2010) Detection of a novel reassortant epizootic hemorrhagic disease virus (EHDV) in the USA containing RNA segments derived from both exotic (EHDV-6) and endemic (EHDV-2) serotypes. J Gen Virol 91: 430–439. 19828758 10.1099/vir.0.015651-0

[60] Attoui H, Billoir F, Cantaloube JF, Biagini P, de Micco P, et al (2000) Strategies for the sequence determination of viral dsRNA genomes. J Virol Methods 89: 147–158. 10996648 10.1016/s0166-0934(00)00212-3

[61] Maan S, Rao S, Maan NS, Anthony SJ, Attoui H, et al (2007) Rapid cDNA synthesis and sequencing techniques for the genetic study of bluetongue and other dsRNA viruses. J Virol Methods 143: 132–139. 17433453 10.1016/j.jviromet.2007.02.016

[62] Larkin MA, Blackshields G, Brown NP, Chenna R, McGettigan PA, et al (2007) Clustal W and Clustal X version 2.0. Bioinformatics 23: 2947–2948. 17846036 10.1093/bioinformatics/btm404

[63] Katoh K, Asimenos G, Toh H (2009) Multiple alignment of DNA sequences with MAFFT. Methods Mol Biol 537: 39–64. 19378139 10.1007/978-1-59745-251-9_3

[64] Xia X, Xie Z (2001) DAMBE: software package for data analysis in molecular biology and evolution. J Hered 92: 371–373. 11535656 10.1093/jhered/92.4.371

[65] Tamura K, Peterson D, Peterson N, Stecher G, Nei M, et al (2011) MEGA5: Molecular Evolutionary Genetics Analysis using Maximum Likelihood, Evolutionary Distance, and Maximum Parsimony Methods. Mol Biol Evol 28: 2731–2739. 21546353 10.1093/molbev/msr121 PMC3203626

[66] Saitou N, Nei M (1987) The neighbor-joining method: a new method for reconstructing phylogenetic trees. Mol Biol Evol 4: 406–425. 3447015 10.1093/oxfordjournals.molbev.a040454

