# Supplementary material for: Full Genome Sequencing of Corriparta Virus, Identifies California Mosquito Pool Virus as a Member of the Corriparta virus Species
Source: PLoS One. 2013 Aug 27;8(8):e70779. doi: 10.1371/journal.pone.0070779 (PMC3754974; doi:10.1371/journal.pone.0070779)
Supplement: Table S1 — Nucleotide accession numbers for sequences used in phylogenetic analysis. (DOCX) [file pone.0070779.s001.docx]

**Supplementary data**

**Table S1: List of nucleotide accession numbers used for sequence and phylogenetic analysis**

| **Species** | **Virus** | **VP1** | **OC1** | **T2** | **CaP** | **NS1** | **OC2** | **T13** | **NS2** | **VP6** | **NS3** |
| --- | --- | --- | --- | --- | --- | --- | --- | --- | --- | --- | --- |
| ***Bluetongue virus*** | **BTV8 (NET2006/04)** | AM498051 | AM498052 | AM498053 | AM498054 | AM498055 | AM498056 | AM498057 | AM498058 | AM498059 | AM498060 |
|  | **BTV-25** | GQ982522 | EU839840 | GQ982523 | GQ982524 | EU839841 | EU839842 | EU839843 | EU839844 | EU839845 | EU839846 |
|  | **BTV-26**  **(KUW2010/02)** | JN255156 | HM590642 | HM590643 | JN255157 | JN255158 | JN255159 | HM590644 | JN255160 | JN255161 | JN255162 |
|  | **Other BTV sequences used** | GQ506536,  GU390658 | --- | DQ186822  DQ186826 | --- | --- | --- | L11723,  GQ506542 | --- | --- | --- |
| ***African horse sickness virus*** | **AHSV-1**  **(HS29/62)** | FJ183364 | FJ183365 | FJ183366 | FJ183367 | FJ183368 | FJ183369 | FJ183370 | FJ183371 | FJ183372 | FJ183373 |
| ***Epizootic haemorrhagic disease virus*** | **EHDV-1w**  **(USA1955/01)** | AM744977 | AM744978 | AM744979 | AM744980 | AM744981 | AM744982 | AM744983 | AM744984 | AM744985 | AM744986 |
|  | **Other EHDV sequences used** | AM744987 | --- | AM744999  AM745029 | --- | --- | --- | AM744993  AM745003 | --- | --- | --- |
| ***Equine encephalosis virus*** | **EEV**  **(HS103/06)** | FJ183384 | FJ183385 | FJ183386 | FJ183387 | FJ183388 | FJ183389 | FJ183391 | FJ183390 | FJ183392 | FJ183393 |
| ***Eubenangee viurs*** | **EUBV**  **(AUS1963/01)** | JQ070376 | JQ070377 | JQ070378  AF530087^*^ | JQ070379 | JQ070380 | JQ070381 | JQ070382 | JQ070383 | JQ070384 | JQ070385 |
|  | **TILV**  **(AUS1978/03)** | JQ070366 | JQ070367 | JQ070368 | JQ070369 | JQ070370 | JQ070371 | JQ070372 | JQ070373 | JQ070374 | JQ070375 |
| ***Palyam virus*** | **CHUV** | AB018086 | AB014725 | AB014728 | AB018087 | AB018089 | AB014726 | AB014727 | AB018090 | AB018088 | AB018091 |
|  | **DAGV**  **(B8112)** | --- |  | AF530085 | --- | --- | --- | --- | --- | --- | --- |
| ***Umatilla virus*** | **UMAV**  **(USA1969/01)** | HQ842619 | HQ842621 | HQ842620 | HQ842623 | HQ842622 | HQ842624 | HQ842626 | HQ842625 | HQ842627 | HQ842628 |
|  | **SLOV** | NC_012754 | --- | NC_012755 | --- | --- | --- | --- | --- | --- | --- |
| ***Peruvian horse sickness virus*** | **PHSV** | DQ248057 | DQ248059 | DQ248058 | DQ248060 | DQ248064 | DQ248061 | DQ248063 | DQ248065 | DQ248062 | DQ248066 |
| ***Yunnan orbivirus*** | **YUOV** | AY701509 | AY701511 | AY701510 | AY701512 | AY701513 | AY701514 | AY701516 | AY701515 | AY701517 | AY701518 |
|  | **MPOV** | --- | EF591621 | EF591620 | --- | --- | --- | --- | --- | --- | --- |
| ***Great Island virus*** | **GIV**  **(CanAr 42)** | HM543465 | HM543469 | HM543466 | HM543467 | HM543468 | HM543470 | HM543471 | HM543472 | HM543473 | HM543474 |
|  | **BRDV** | --- | --- | M87875 | --- | X82599 | M58030 | M87876 | --- | --- | M83197 |
|  | **LIPV**  **(CzArLip 91)** | HM543475 | --- | HM543476 | --- | --- | HM543477 | --- | --- | --- | --- |
|  | **TRBV** | HM543478 | --- | HM543479 | --- | --- | HM543480 | --- | --- | --- | --- |
|  | **KEMV**  **(EgAn 1169-61)** | HM543481 | --- | HM543482 | --- | --- | HM543483 | --- | --- | --- | --- |
| ***St Croix river virus*** | **SCRV** | NC_005997 | NC_005998 | NC_005999 | NC_006000 | NC_006002 | NC_006001 | NC_006004 | NC_006003 | NC_006005 | NC_006006 |
| ***Corriparta virus*** | **CORV** | --- | --- | AF530086^*^ | --- | --- | --- | --- | --- | --- | --- |
| ***Warrego virus*** | **WARV**  **(Ch 9935)** | --- | --- | AF530083^*^  EF213555^*^ | --- | --- | --- | --- | --- | --- | --- |
| ***Wallal virus*** | **WALV**  **(Ch 12048)** | --- | --- | AF530084^*^ | --- | --- | --- | --- | --- | --- | --- |
| ***Wongorr virus*** | **WGRV**  **(V5080, V195, V199, mrm13443)** | --- | --- | U56989^*^,  U56990^*^,  U56991^*^,  U56992^*^ | --- | --- | --- | --- | --- | --- | --- |
|  | **PARV** | --- | --- | U56993^*^ | --- | --- | --- | --- | --- | --- | --- |
|  | **PIAV** | --- | --- | U56994^*^ | --- | --- | --- | --- | --- | --- | --- |
| **Pata virus** | **PATAV**  **(CAF1968/01)** | JQ070386 | JQ070387 | JQ070388 | JQ070389 | JQ070390 | JQ070391 | JQ070393 | JQ070392 | JQ070394 | JQ070395 |
| **California mosquito pool virus** | **CMPV** | --- | --- | EU789391^*^ | EU789392^*^ | --- | EU789393^*^ | EU789394^*^ | --- | EU789395^*^ | --- |

^*^only partial sequences are available. Orbivirus species for which full genome sequences are available are highlighted in yellow color and for which partial sequences are available are highlighted in light green colour. Pata virus (represented in grey colour) represents novel species in the genus *Orbivirus* (Belaganahalli et al., 2012). **Pol**=Polymerase, **OC1**=Outer capsid protein 1 (VP2 of BTV), **T2**=Inner core protein (T2 symmetry), **Cap**= Capping enzyme, **Tup**=Tubule forming protein or Tubular protein (NS1), **OC2**=Outer capsid protein 2 (VP5 of BTV), **T13**=Outer core protein (T13 symmetry), **ViP**=Viral inclusion body protein (NS2), **Hel**=Helicase protein.
